# An assessment of survival outcomes among ovarian cancer patients at the National and Referral Hospital in Kenya

**DOI:** 10.1002/cnr2.1986

**Published:** 2024-02-13

**Authors:** Diana Bironga Mayenga, Amsalu Degu

**Affiliations:** ^1^ School of Pharmacy and Health Sciences, Department of Pharmaceutics and Pharmacy Practice United States International University‐Africa Nairobi Kenya

**Keywords:** Kenyatta National Hospital, ovarian cancer, survival outcomes

## Abstract

**Background:**

Ovarian cancer has been shown to have poor survival outcomes attributed to late presentation. In Kenya, information on the survival outcomes of ovarian cancer patients is scarce. Therefore, the objective of this study was to examine the survival outcomes among patients with ovarian cancer treated at Kenyatta National Hospital (KNH).

**Aims:**

A hospital‐based retrospective cohort study was performed at KNH to examine the survival outcomes of 112 ovarian cancer patients. The study employed a structured data abstraction tool to acquire patients' relevant socio‐demographic and clinical characteristics from the patient's medical records. The data obtained were analyzed using SPSS version 29.0 statistical software. Kaplan–Meier and Cox regression analyses were used to determine the survival outcome and predictors of mortality among ovarian cancer patients, respectively.

**Methods and results:**

The mean age of the patients in this study was 51.28 ± 14.24 years. Most patients (59.8%) had evidence of distant metastasis during the follow‐up period. One‐third (33%) of patients were deceased. The mean‐cancer‐specific survival time among the study participants was 40.0 ± 3.0 months. The 5‐year survival rate was 44%, with most patients experiencing disease progression during the last follow‐up. Combination therapy (*p* < .001) was the only statistically significant predictor of mortality in ovarian cancer patients.

**Conclusion:**

The study found that the 5‐year survival rate among ovarian cancer patients was poor, with most patients experiencing disease progression during the last follow‐up period.

## 1 INTRODUCTION

Among females, ovarian cancer ranks as the third most prevalent gynecological malignancy, following cervical and uterine cancer.^1^ Ovarian cancer constituted 1.6% of 18.1 million new cancer diagnoses and 1.9% of the 9.6 million global cancer‐related fatalities.^2^ In contrast to breast cancer, ovarian cancer has a lower incidence rate, yet it carries a higher mortality rate. The mortality rate due to ovarian cancer is estimated to significantly increase by the year 2040.^1^

In the African continent, approximately 25 000 new cases and 17 000 fatalities of ovarian cancer were recorded in 2020,^1^ with a mortality‐to‐incidence ratio of 0.7, making ovarian cancer among the top ten lethal cancers.^3^ A recent study in Sudan showed that most ovarian cancer patients presented with an advanced stage of the disease, resulting in shorter survival times.^4^ Nonetheless, another study showed that early detection does not improve overall survival.^5^ In contrast, other studies have reported that ovarian cancer survival can be improved by early detection.^6^, ^7^

The emergence of newer treatments in recent years, including intraperitoneal chemotherapy and treatment with antiangiogenic and targeted chemotherapies, has improved survival rates.^8^ Despite this, the 5‐year survival rates of patients with ovarian cancer remain low.^4^, ^9^ Therefore, achieving the desired goal of improved survival rates remains challenging. Moreover, there is a paucity of adequate data on the survival outcomes of ovarian cancer patients in Kenya. Thus, the primary objective of this study was to evaluate the survival outcomes experienced by patients with ovarian cancer receiving treatment at Kenyatta National Hospital (KNH).

## 2 MATERIALS AND METHODS

### 2.1 Study design

A hospital‐based retrospective cohort study was employed to evaluate the survival outcomes and the associated factors among ovarian cancer patients who received treatment at the KNH from January 1st, 2017 until December 31st, 2021.

### 2.2 Study setting and period

This study was conducted for 6 months (October 1st, 2022 to March 31st, 2023) at KNH. The hospital was founded in 1901 and is located along Hospital Road in Nairobi, Kenya. It is one of the largest referral and teaching hospitals in Kenya.

### 2.3 Study population

The study population included all eligible adult ovarian cancer patients who had been treated at the KNH from January 1st, 2017 until December 31st, 2021.

## 3 ELIGIBILITY CRITERIA

### 3.1 Inclusion criteria

Medical records of patients aged 18 years and above with a confirmed diagnosis of ovarian cancer treated from January 1st, 2017 until December 31st, 2021.

Patients who had comprehensive medical records detailing their treatment regimen, cancer stage, and details on their diagnosis.

Patients who had a complete interval imaging results to assess the response of the tumor.

### 3.2 Exclusion criteria

Patients with incomplete medical records on their treatment regimen, cancer diagnosis, and cancer stage were excluded from the study.

**FIGURE 1:**
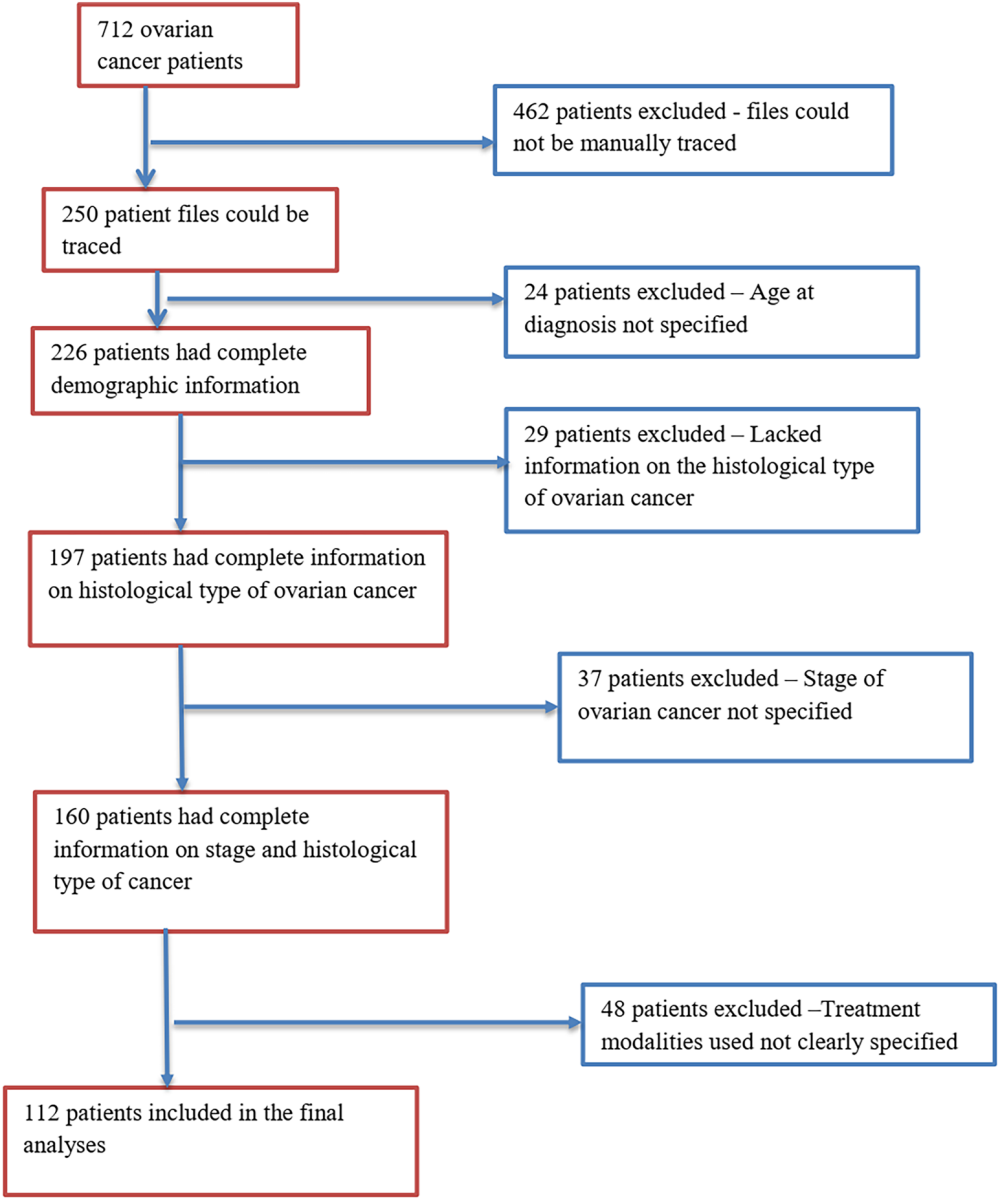
Inclusion and exclusion criteria among ovarian cancer patients.

The details of the eligibility criteria are stated in Figure 1.

### 3.3 Sample size determination

The health records department at KNH identified ovarian cancer cases treated at their facility through an electronic search by use of the International Classification of Diseases, 10th Revision (ICD‐10) Diagnosis Code C56, which is specific for malignant neoplasm of the ovary. The files at the facility registered under the code were filtered by the system to include ovarian cancer cases according to the eligibility criteria and 712 cases were identified. The identified files were manually extracted from the file's storage, of which only 250 files could be traced, and of the total, only 112 files had complete relevant information required for the study. In this study, therefore, 112 eligible patients comprised the study's sample size (Figure 1).

### 3.4 Data collection tools and procedures

A data abstraction tool was used to collect data from the medical records of patients. The data abstraction tool contained essential information, such as socio‐demographic factors of the patients, clinical characteristics, and survival outcome measuring parameters. The structured data abstraction tool was designed in reference to previously published survival outcome studies done in our setting with some modifications.^10^, ^11^, ^12^, ^13^ A pretest was conducted on 5% of the total sample size. Before employing the data collection tool in the main study, all necessary adjustments were made. The Health Information Department of KNH was consulted to obtain the patient's medical records. The research assistant and principal investigators conducted a manual extraction of data from the patient's medical records. Subsequently, a comprehensive review was performed among them to ensure data completeness and accuracy, with any discrepancies meticulously examined and resolved through adjudication. Relevant information was assessed, including the patient's socio‐demographics, treatment history, time of death, length of survival, and response to therapy. Mortality and disease progression were assessed as outcomes. The data obtained were then reviewed using appropriate data analysis tools.

### 3.5 Data analysis

The data collected were entered and analyzed using SPSS version 29.0 software. Socio‐demographic factors, such as patient age, were described using mean and standard deviation. On the other hand, marital status, level of education, and employment status were presented using frequency and percent. Clinical characteristics, treatment regimens and treatment outcomes including tumor size after treatment, patient status, and the presence of distant metastasis among others, were also presented through frequency and percentage tables. For the tumor size after treatment, the baseline for tumor size measurement was established using computerized tomography (CT) scan results. The increase or decrease in tumor size was assessed by comparing posttreatment CT scans with the baseline scans.

The mean cancer‐specific survival was calculated from the months between the initial diagnosis and death or last follow‐up. Metastasis‐free survival was determined from the months between the initial diagnosis and the first occurrence of radiologic metastasis. Cancer‐specific survival after metastasis was computed from the months between the first occurrence of radiologic metastasis and death or last follow‐up. The data were computed by calculating an average of the months.

Survival outcomes for ovarian cancer patients were assessed through Kaplan–Meier analysis. The 5‐year survival rate analysis focused on ovarian cancer patients who survived for at least 5 years from the initial diagnosis. Cox regression analysis, was conducted to estimate predictors of mortality. In bivariate Cox regression analyses, independent variables (e.g., age, cancer stage, histological type, comorbidity, distant metastasis, and treatment modalities) were compared with the outcome variable (censored or mortality). In multivariate Cox regression analyses, the independent variables (stage of cancer, distant metastasis, and type of treatment regimens) were collectively assessed against the outcome variable to identify predictors of mortality. Variables with a *p*‐value above .10 in the bivariate analyses (age, histological type, and comorbidity) were excluded from multivariable analyses.

## 4 RESULTS

### 4.1 Socio‐demographic characteristics

The mean age of the patients in this study was 51.28 ± 14.24 years. Regarding the employment status, most of them were unemployed (54.5%), whereas 38.4% were private employees, 3.6% were retired, and 3.6% were employed (Table 1).

**TABLE 1:** Socio‐demographic characteristics of the study participants (*N* = 112).

Variables	Frequency	Percent (%)
Age		
<60 years	73	65.2
≥60 years	39	34.8
Marital status		
Divorced	2	1.8
Married	63	56.3
Single	41	36.6
Widowed	6	5.4
Level of education		
Illiterate	9	8.0
Primary	46	41.1
Secondary	41	36.6
Tertiary	16	14.3
Employment status		
Employed	4	3.6
Private employee	43	38.4
Retired	4	3.6
Unemployed	61	54.5

*Note*: Illiterate (lack basic formal education), primary (class 1–8), secondary/high school (class 9–12), tertiary/postsecondary (technical and vocational education and training, colleges, and universities).

### 4.2 Clinical characteristics

Moreover, the median follow‐up time of the patients in this study was 13.5 months and the maximum and minimum follow‐up time was 63 months and 1 month, respectively. The predominant histological type of ovarian cancer, epithelial ovarian cancer, was observed in 82.1% of the patients, followed by sex cord‐stromal ovarian cancer (12.5%) and germ cell cancer (5.4%). Most patients (50.9%) presented with stage IV disease at diagnosis. The most prevalent co‐morbidity was hypertension, accounting for 29.5% of all patients (Table 2).

**TABLE 2:** Clinical characteristics of ovarian cancer patients (*N* = 112).

Variable	Frequency	Percent (%)
Histological type		
Epithelial	92	82.1
Germ cell	6	5.4
Sex cord‐stromal	14	12.5
Stage of cancer		
Stage I	23	20.5
Stage II	13	11.6
Stage III	19	17.0
Stage IV	57	50.9
Comorbidity		
Present	42	37.5
Absent	70	62.5
Number of comorbidities		
0	71	63.4
1	28	25.0
2	10	8.9
3	3	2.7
Types of comorbidity		
Hypertension	33	29.5
Chronic kidney disease	2	1.8
Deep vein thrombosis	4	3.6
Epilepsy	1	0.9
Congestive heart failure	1	0.9
Diabetes	9	8.0
Goiter	1	0.9
Retroviral disease	6	5.4
Stroke	1	0.9

**FIGURE 2:**
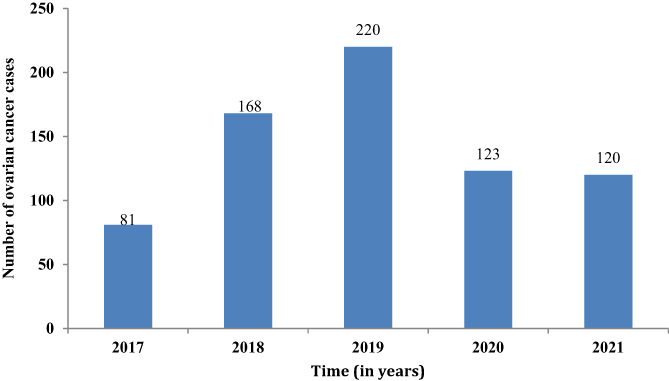
Trend of diagnosis among ovarian cancer patients.

The standard treatment of the majority of the patients (65.2%) was surgery and chemotherapy, followed by surgery (28.6%). Most patients (36.6%) underwent six cycles of chemotherapy to manage their malignancy (Table 3). On the trend of diagnosis, it was observed that most patients were diagnosed in year 2019 (220), out of the total 712 cases over the study period (Figure 2).

**TABLE 3:** Treatment regimen of the study participants (*N* = 112).

Variable	Frequency	Percent (%)
Treatment modalities		
Chemotherapy only	5	4.5
Radiotherapy + chemotherapy	1	0.9
Surgery only	32	28.6
Surgery + chemotherapy	73	65.2
Surgery + radiotherapy	1	0.9
Type of chemotherapy		
Adjuvant platinum and taxane‐based regimen	53	47.3
Adjuvant platinum‐based regimen	15	13.4
Neo‐adjuvant platinum and taxane‐based regimen	11	9.8
No chemotherapy	33	29.5
Number of chemotherapy cycles		
0	33	29.4
1	12	10.7
2	2	1.8
3	4	3.6
4	11	9.8
5	5	4.5
6	41	36.6
9	1	0.9
12	3	2.7

### 4.3 Treatment outcomes

Most patients (59.8%) had evidence of distant metastasis during the follow‐up period, whereas 40.2% had no evidence of distant metastasis. Two‐thirds (67.0%) of patients were censored, and 33% died. For most patients (42.9%), the CA‐125 levels significantly increased during the follow‐up period (Table 4). The mean‐cancer‐specific survival time among the study participants was 40.0 ± 3.0 months. On the other hand, the metastasis‐free survival and cancer‐specific survival after metastasis were 15.5 ± 4.3 months and 16.7 ± 2.3 months, respectively (Table 5). The present study showed that the 5‐year survival rate of ovarian cancer patients was 44%.

**TABLE 4:** Treatment outcomes of ovarian cancer patients (*N* = 112).

Variable	Frequency	Percent (%)
Tumor size after treatment		
Decreased	55	49.1
No change	9	8.0
Progressed	48	42.9
Distant metastasis during follow‐up period		
Yes	67	59.8
No	45	40.2
Patient status after treatment		
Dead	37	33.0
Censored	75	67.0
Status of the response of cancer during the last follow‐up period		
Complete response	28	25.0
Disease progression	56	50.0
No response	5	4.5
Partial response	19	17.0
Stable disease	4	3.6
CA‐125 level in the last follow‐up period		
No change	23	20.5
Significantly increased	48	42.9
Significantly reduced	41	36.6

**TABLE 5:** Survival parameters among the study participants.

Survival parameter	Mean (months) ± standard error
Cancer‐specific survival	40.0 ± 3.0
Metastasis‐free survival	15.5 ± 4.3
Cancer‐specific survival after metastasis	16.7 ± 2.3

### 4.4 Predictors of mortality

**FIGURE 3:**
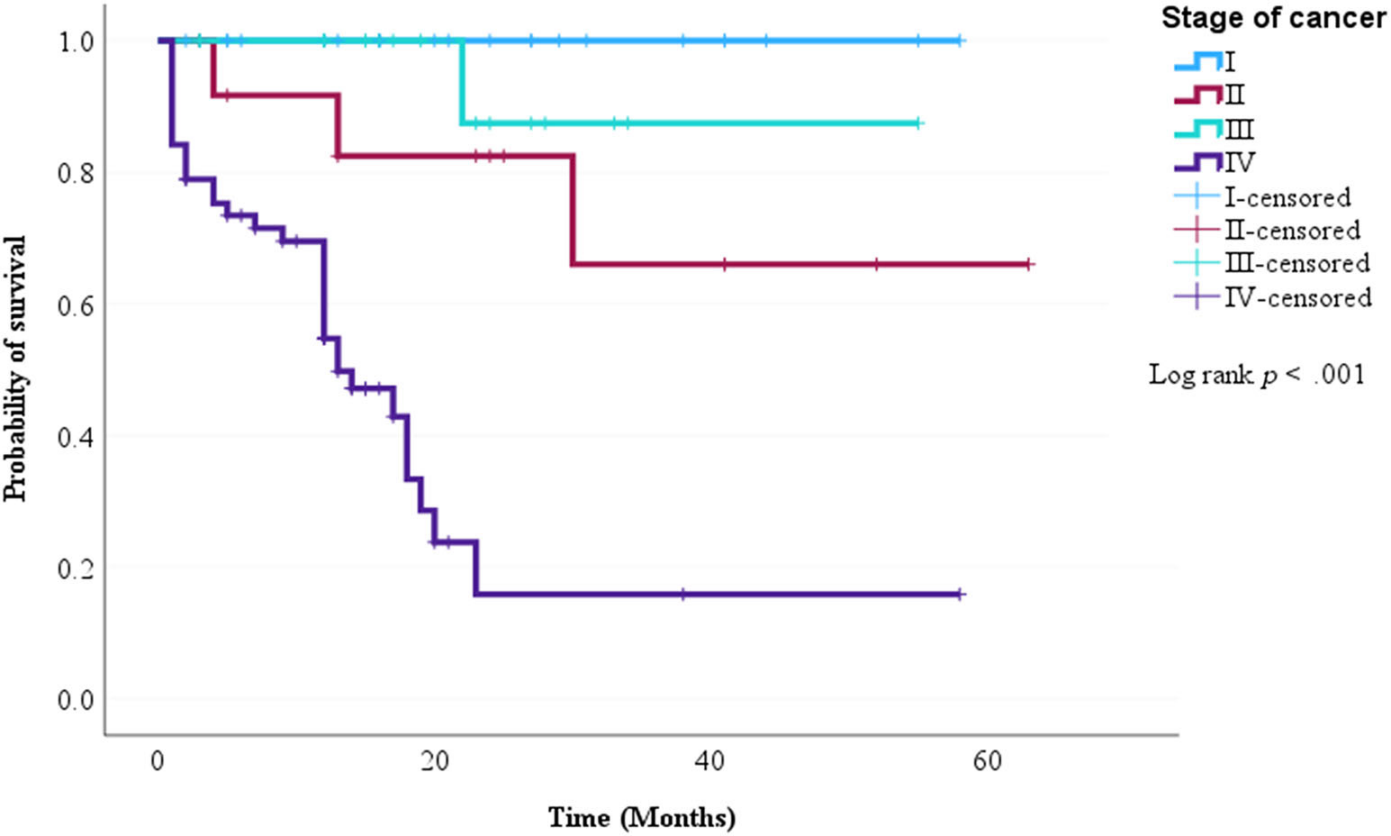
Kaplan–Meier survival curve of ovarian cancer patients based on tumor stage.

**FIGURE 4:**
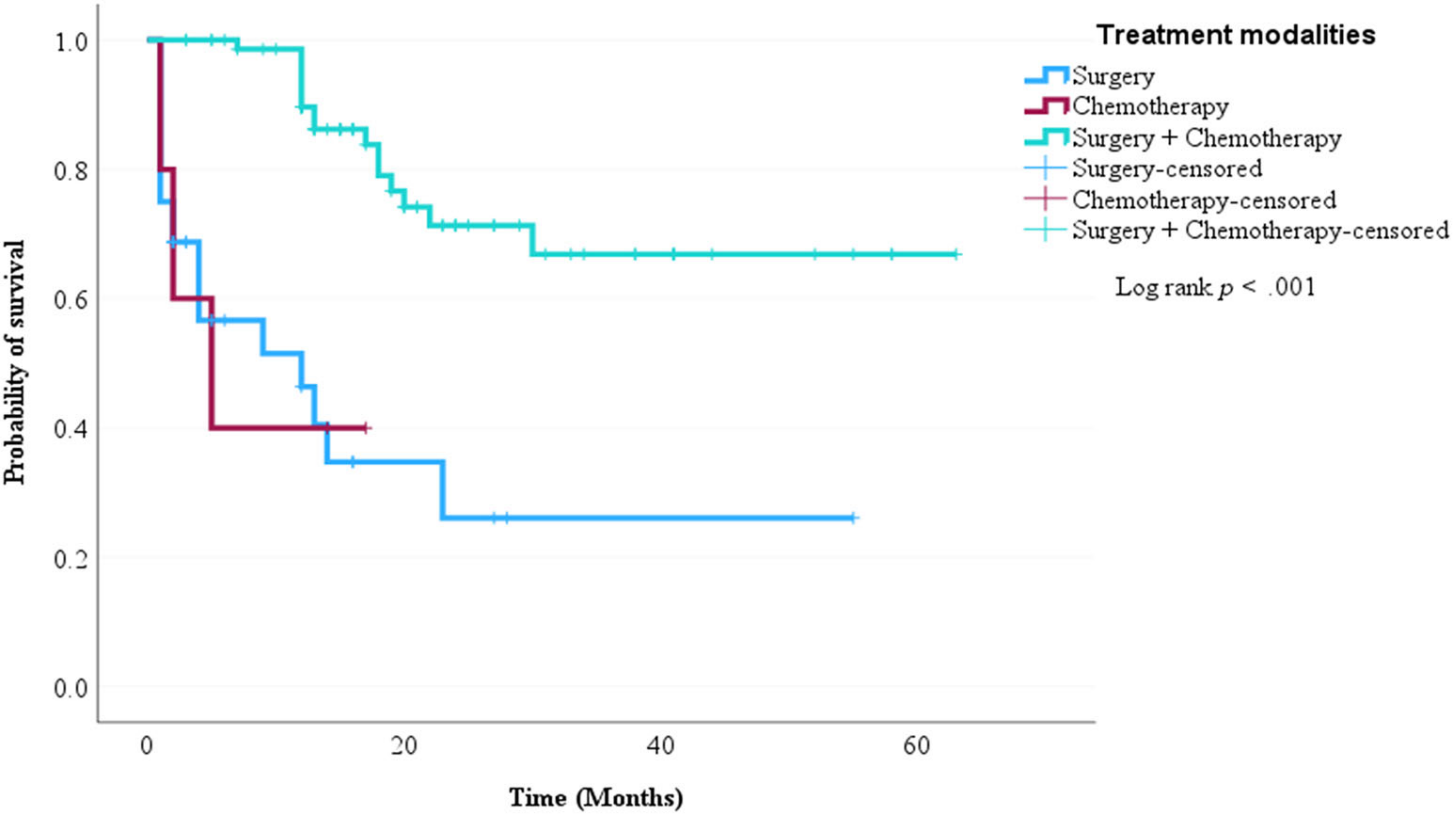
Kaplan–Meier survival curve of ovarian cancer patients based on treatment regimen.

The Cox regression was used to perform bivariate and multivariate analyses to investigate the association between the independent variables and the risk of death. The variables used were age, cancer stage, histological type, co‐morbidity, distant metastasis, and treatment regimens. Combination therapy was the only statistically significant predictor of mortality in ovarian cancer patients (Table 6). Nonetheless, in the log‐rank test, there was a significant difference in survival rate among different stages of cancer and treatment regimens (Figures 3 and 4).

**TABLE 6:** Predictors of mortality among ovarian cancer patients.

	Bivariate analysis		Multivariate analysis	
Variable	CHR (95% CI)	*p*‐value	AHR (95% CI)	*p*‐value
Age				
<60 years	1	1		
≥60 years	1.3 (0.7–2.6)	.387		
Stage of cancer			1	1
Early stage (I and II)	1	1	1.8 (0.2–19.1)	.611
Advanced stage (III and IV)	8.6 (2.6–28.2)	<.001*		
Histological type of cancer				
Epithelial	1	1		
Germ cell	0.4 (0.05–2.6)	.312		
Sex‐cord stromal	0.5 (0.2–1.6)	.228		
Comorbidity				
Absent	1	1		
Present	1.2 (0.6–2.4)	.556		
Distant metastasis			1	1
No	1	1	7.2 (0.9–55.7)	.057
Yes	10.8 (3.7–31.3)	<.001*		
Type of treatment regimen			1	1
Surgery	1	1	1.2 (0.3–4.3)	.802
Chemotherapy	1.1 (0.3–3.7)	.913	0.2 (0.1–0.4)	<.001*
Combination therapy	0.2 (0.1–0.4)	<.001*		

*Note*: Adjusted for age, stage of cancer, histological type of cancer, comorbidity, distant metastasis, and type of treatment regimen. Combination therapy (surgery and chemotherapy).

Abbreviations: AHR, Adjusted hazard ratio; CHR, Crude hazard ratio.

* Statistically significant with *p*‐value <.05.

## 5 DISCUSSION

This retrospective study examined survival outcomes and factors linked to ovarian cancer patients at KNH. In terms of the occurrence pattern, the results in this study indicated that most cases of ovarian cancer were reported in patients below 60 years (65%) with a mean age of 51.28 ± 14.24 years. In Taiwan, the mean age at diagnosis was 52.8 ± 11.2 years.^14^ Moreover, previous research has found that the median age at diagnosis ranges from 50 to 79 years.^15^ A recent study reported that most ovarian cancer cases were above 50 years.^16^ This is consistent with the findings in Nigeria, where ovarian cancer was most prevalent around the fifth decade of life.^17^ Although several studies have reported that ovarian cancer diagnosed at a younger age has a better prognosis,^18^, ^19^, ^20^ other researchers have argued that age is not a standalone prognostic factor.^21^

Epithelial, stromal, or germ‐cell tumors account for most benign and malignant ovarian cancers. Various studies have reported that in developed countries, up to 90% of all malignant ovarian cancers have an epithelial origin, 5%–6% of the tumors are sex‐cord stromal tumors and 2%–3% originate from the germ cells.^18^, ^22^ Similarly, this study found that epithelial ovarian cancer was the most common histological type, accounting for 82.1% of all ovarian cancer cases.

Previous studies have reported that the stage at detection has a considerable impact on the survival of ovarian cancer patients.^14^, ^23^ Most ovarian cancer patients present with an advanced stage of the disease due to the insidious onset of nonspecific symptoms and a lack of timely and proper screening.^6^ However, a study conducted in Taiwan reported that a relatively high percentage of the patients (44.4%) had an early‐stage disease, which could be attributed to the ease of access to undergo early screening.^23^ In the present study, the majority of the patients (50.9%) presented with stage IV disease at diagnosis, which can be ascribed to poor health‐seeking habits as a result of the study participants' low socioeconomic status and level of education.^24^

A third (37.5%) of the total study population presented with co‐morbidities, with hypertension being the most common type (29.5%), followed by diabetes (8.0%). In general, there is an established correlation between hypertension and an elevated risk of developing cancer. A recent study suggested that hypertension causes dysregulation of apoptosis, therefore, increasing cancer risk.^25^ Additionally, another study hypothesized that elevated angiotensin II levels in hypertensive patients can stimulate the synthesis of vascular endothelial growth factor, which augments cancer‐related angiogenesis.^26^ A cohort study on cancer patients reported hypertension as the most common co‐morbidity.^27^ A recent study conducted in Saudi Arabia reported diabetes mellitus and hypertension as the most common co‐morbidities affecting ovarian cancer patients since each of them accounted for 39.5% of the total study population.^28^ Another study reported hypertension (11%–26%), cardiovascular disease (4.5%–12%), and diabetes (2.5%–8.3%) as the most prevalent comorbidities in ovarian cancer patients.^29^

The recommended standard treatment guideline for ovarian cancer involves surgery followed by platinum‐based combination chemotherapy.^30^ Similarly, patients who received adjuvant combination chemotherapy of a taxane‐based regimen were reported to have considerably improved overall survival than those who received non‐taxane‐based chemotherapy regardless of the stage of cancer.^14^ Another study also reported similar results in ovarian cancer patients who received the taxane‐based combination chemotherapy had significantly improved post‐recurrence survival.^31^ In our setting, two‐thirds (65.2%) of the patient population were treated with surgery and chemotherapy. The most common chemotherapy regimen administered was adjuvant cisplatin and paclitaxel. Moreover, most patients received taxane‐based combination chemotherapy composed of a platinum drug such as cisplatin or carboplatin with either paclitaxel or docetaxel, hence the significant reduction of tumor size after treatment observed in close to half (49.1%) of the study population.

The disease progression and treatment efficacy in ovarian cancer patients are monitored using changes in CA‐125 levels.^32^ Various studies have found a relationship between increasing CA‐125 levels posttreatment with an increased risk of ovarian cancer recurrence.^33^, ^34^ Consequently, the increasing CA‐125 levels in the first months posttreatment may indicate resistance to platinum‐based chemotherapy.^35^ In the present study, for most patients (42.9%), the CA‐125 levels significantly increased during the follow‐up period, with 36.6% having the CA‐125 levels significantly decreased and 20.5% experiencing no change in the CA‐125 levels posttreatment.

In the present study, the 5‐year survival rate of ovarian cancer patients was 44%. This finding is similar to a study conducted in Canada, which reported a 5‐year survival rate of 44%.^18^ Another European cohort study reported 5‐year survival rates ranging from 26% to 51%.^23^ A study conducted in Sudan reported that the 5‐year cumulative survival rate was 38% for all histological types of ovarian cancer.^4^ The relatively low survival rates reported among ovarian cancer patients could be due to most of them presenting with an advanced stage of the disease, as was observed in our setting, which could be attributed to the delayed onset of symptoms and the lack of health‐seeking behavior among ovarian cancer patients. Additionally, with the advanced tumor stage, the tumor has metastasized to other organs in the body, and the likelihood of recurrence of the tumor is high despite initial treatment.

Several variables that have been associated with predicting survival in ovarian cancer include the stage at detection, histologic subtype, age, tumor grade, postoperative residual tumor, response to chemotherapy, co‐morbidities, and pretreatment serum concentrations of CA‐125.^9^, ^36^ Age, histological type, stage of cancer, distant metastasis, co‐morbidities, and treatment regimen were the factors considered in our setting. Of these variables, the only significant factor influencing survival outcomes was combination therapy (surgery and chemotherapy only) among ovarian cancer patients.

### 5.1 Limitations of the study

The study was retrospective in design; therefore, it relied on the effective documentation of medical records, which was lacking, as evidenced by a high percentage of missing data.

## 6 CONCLUSION

The 5‐year survival rate among ovarian cancer patients was poor, with most patients experiencing disease progression during the last follow‐up period.

## AUTHOR CONTRIBUTIONS

**Diana Bironga Mayenga:** Conceptualization (equal); data curation (equal); formal analysis (equal); funding acquisition (equal); investigation (equal); methodology (equal); project administration (equal); resources (equal); software (equal); supervision (equal); validation (equal); visualization (equal); writing – original draft (equal); writing – review and editing (equal). **Amsalu Degu:** Conceptualization (equal); data curation (equal); formal analysis (equal); funding acquisition (equal); investigation (equal); methodology (equal); project administration (equal); resources (equal); software (equal); supervision (equal); validation (equal); visualization (equal); writing – original draft (equal); writing – review and editing (equal).

## FUNDING INFORMATION

This study was conducted without any funding.

## CONFLICT OF INTEREST STATEMENT

The authors have stated explicitly that there are no conflicts of interest in connection with this article.

## ETHICS STATEMENT

Upon receiving approval from the University of Nairobi/Kenyatta National Hospital Ethics and Research Committee, the process of actual data collection commenced (Reference number: UP755/09/2022). Patient data privacy was maintained by using their initials and unique patient identification numbers.

## PATIENT CONSENT STATEMENT

Because of the study's retrospective nature, the Ethics Committee granted waivers for informed consent.

## Data Availability

The datasets used and/or analyzed during the current study will be obtained from the corresponding author of this project.
